# Antimicrobial Lock Therapy: A Strategy for Managing Catheter-Related Bacteremia

**DOI:** 10.3390/antibiotics14050461

**Published:** 2025-04-30

**Authors:** Firdevs Aksoy, Hanife Nur Karakoc Parlayan, Gulter Oncu Kurutas, Gurdal Yilmaz

**Affiliations:** Department of Infectious Disease and Clinical Microbiology, Faculty of Medicine, Karadeniz Technical University, 61080 Trabzon, Türkiye; faslanaksoy@ktu.edu.tr (F.A.); gulteroncu@ktu.edu.tr (G.O.K.); gurdalyilmaz@ktu.edu.tr (G.Y.)

**Keywords:** catheter-related bloodstream infections, lock therapy, hemodialysis, long-term catheters

## Abstract

Objectives: This study aims to evaluate the use and efficacy of antibiotic-lock therapy (ALT) in the management of catheter-related bloodstream infections (CRBSIs), focusing on its impact on infection resolution, catheter retention, and clinical outcomes. Methods: Patients aged ≥18 years diagnosed with CRBSIs who had long-term indwelling catheters and for whom catheter replacement posed clinical challenges were enrolled in the retrospective study from January 2019 to December 2024. Participants were divided into two groups based on treatment: Group 1 received intravenous (IV) antibiotics combined with antibiotic-lock therapy (ALT), while Group 2 received IV antibiotics alone. Patient demographics, pathogen distribution, administered antibiotic regimens, duration of treatment, laboratory parameters, clinical outcomes, and mortality rates were evaluated. Results: A total of 54 patients were included, of whom 42.6% were female, and the mean age was 66.3 ± 15.4 years. Group 1 comprised 50% of the study population. The median treatment duration was 14 days. The most common pathogen was *Coagulase-negative staphylococci*, and 33.3% of CRBSIs were caused by Gram-negative bacteria (GNB). Group 1 demonstrated lower C-reactive protein levels at treatment 48/72 h of treatment (*p* = 0.013) and a reduced frequency of catheter revision (*p* < 0.0001) compared to Group 2. Overall, ALT achieved a success rate of 88.9%, with success rates of 86% for GNB infections and 90% for Gram-positive bacterial infections. Among patients receiving daily ALT, the success rate was 86%, while those receiving the therapy every three days had a success rate of 90%. Conclusions: Antimicrobial lock therapy can be considered a treatment option for managing CRBSIs, particularly in cases where removal of the implantable catheter is not feasible, allowing for salvage.

## 1. Introduction

Long-term intravascular catheter use has a pivotal role in managing patients with chronic medical conditions, particularly those requiring prolonged vascular access. They are commonly utilized in the treatment of individuals undergoing hemodialysis, parenteral nutrition, and intravenous chemotherapy [1,2]. The use of long-term intravascular catheters has markedly increased, largely driven by the expanding needs of patients. Catheter-related bloodstream infection (CRBSI) is recognized as one of the most significant complications associated with long-term indwelling catheter use [3], contributing to increased morbidity, mortality, and healthcare costs [4]. Among the key mechanisms driving the pathogenesis of these infections, biofilm formation on the catheter surface plays a crucial role. Biofilms, composed of microbial communities embedded within a protective extracellular matrix, not only provide a favorable environment for microbial adherence and proliferation but also contribute to increased resistance against systemic antibiotic therapies. This complex structure often renders conventional treatments insufficient, thereby predisposing patients to persistent and recurrent infections [5,6,7].

Among the strategies developed to manage CRBSIs, antimicrobial lock therapy (ALT), which entails instilling a high-concentrated antibiotic solution into the catheter lumen, has emerged as a promising conservative strategy aimed at eradicating biofilm-associated microorganisms. It is used as an adjunct to systemic antibiotic therapy, especially when removal of the catheter is not possible because of restricted intravenous access or medical indications [2].

ALT can facilitate targeted eradication of biofilms by instilling a high-concentration antibiotic solution (100 to 1000 times the minimum inhibitory concentration (MIC)) into the catheter lumen [5]. When used in combination with systemic antibiotic therapy, ALT offers significant advantages, particularly in cases where catheter removal is technically difficult or associated with increased clinical risk. In such cases, ALT plays a crucial role in achieving complete infection eradication and enabling the long-term retention of the catheter [8,9].

Although ALT has emerged as an effective adjunctive strategy for the management of CRBSIs, it is not universally applicable across all pathogens or clinical scenarios. According to current clinical guidelines, ALT is generally not recommended in infections caused by certain pathogens, such as *Staphylococcus aureus* or *Candida* spp., particularly when the catheter can be safely and easily removed [10,11]. In contrast, it may be considered in selected cases where catheter removal poses significant technical challenges, such as in patients with implanted ports or tunneled catheters used for long-term therapies.

These distinctions play a critical role in the individualization of treatment strategies, taking into account the type of pathogen, the removability of the catheter, and the patient’s overall clinical condition. Moreover, clinical evidence supporting the widespread efficacy of ALT remains limited. Therefore, we hypothesized that the use of ALT in addition to systemic antibiotic treatment would improve clinical outcomes and reduce the need for catheter removal in patients with long-term CRBSIs. The present study aims to evaluate the effectiveness of ALT in the management of CRBSIs and to assess its impact on clinical outcomes, providing insights that may guide future therapeutic approaches.

## 2. Results

Between 1 January 2019 and 31 December 2024, a total of 112 cases with bacteremia were screened. Among these, one had a splenic abscess, two had spondylodiscitis, three had infective endocarditis, one had cholangitis, and two had urinary tract infections as the primary focus and were excluded. Cases with confirmed Candida albicans bloodstream infections (*n* = 3) were excluded from the study due to the absence of detailed treatment data and follow-up information, which prevented outcome assessment. The remaining cases were excluded if they did not have a central venous catheter or if no microbial growth was detected in catheter cultures. A total of 67 CRBSI cases were identified during the study period. A total of 13 cases of catheter-related bacteremia involving short-term catheters were excluded, leaving 54 cases of CRBSIs for analysis. Of these, 27 cases were treated with intravenous (IV) antibiotics combined with ALT, and the remaining 27 cases received IV antibiotics alone.

Among the included cases, 42.6% (*n* = 23) were female, and the mean age was 66.3 ± 15.4 years. Fever was present at hospital admission in 46.3% of cases. The underlying comorbid conditions were observed in 44 (81.5%) patients, with hypertension (HT) as the leading condition (61.1%), followed by diabetes mellitus (DM) (38.9%). The patient demographic and clinical characteristics of the patients are presented in Table 1.

**Figure 1:**
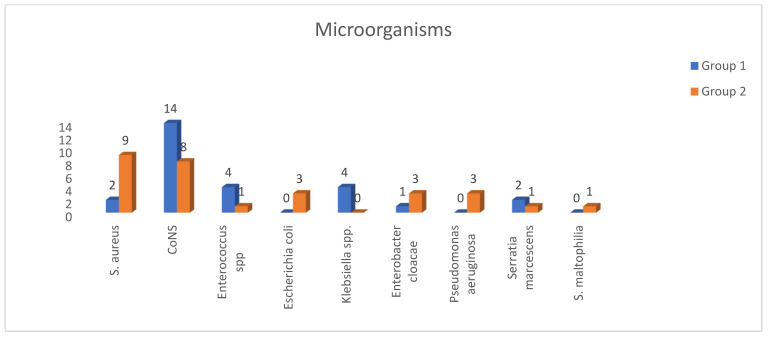
Distribution of pathogens identified in catheter-related bacteremia cases (*n*). *S. aureus*: *Staphylococcus aureus*, CoNS: *Coagulase-negative staphylococci*, *S. maltophilia*: *Stenotrophomonas maltophilia*.

Among 54 cases, we detected CRBSIs, which were classified as polymicrobial in 3 cases (5.6%). Microbiological evaluation revealed Gram-positive bacteria (GPB) in 66.7% of cases, with *Coagulase-negative staphylococci* (CoNS) identified as the most common microorganisms in 22 cases (40.7%); Gram-negative bacteria (GNB) were isolated in 18 infections (33.3%). The distribution of causative microorganisms is presented in Figure 1. The median time from catheter insertion to the CRBSIs was 240 days (range, 5 to 1825 days). Of the catheters, 28 (51.9%) were inserted into the internal jugular vein. Hemodialysis was the most common indication for long-term indwelling catheter placement (51 cases, 94.4%), while other indications included chemotherapy and parenteral nutrition. All 27 cases that received ALT were in the hemodialysis group. In contrast, the three patients with non-dialysis catheter indications were all included in Group 2, which did not receive ALT.

**Figure 2:**
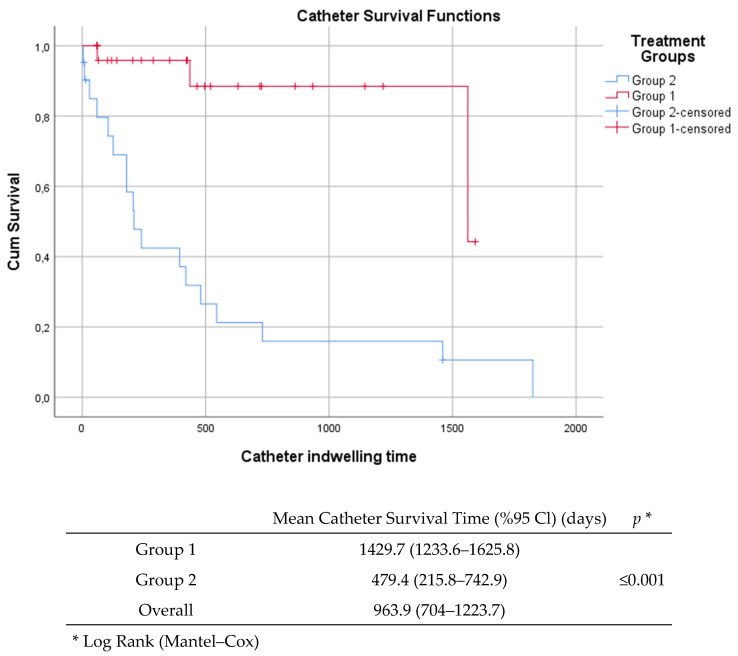
Kaplan–Meier curve of catheter survival.

Details of the antibiotic therapies administered to the patients are presented in Table 2. The median duration of treatment was 14 days (range, 1–33). Some patients received antibiotics from more than one group (11.1%). Glycopeptides were the most frequently used antibiotics in all groups. The median interval between infection diagnosis and follow-up blood culture sampling was 96 h (range, 0 to 360 days). There were no significant differences detected between the groups in terms of treatment duration and catheter days. However, the time to follow-up blood culture sampling was markedly longer in Group 1 (*p* < 0.0001). Kaplan–Meier survival analysis demonstrated significantly longer catheter survival in Group 1 compared to Group 2. The median catheter dwell time was notably longer in the ALT group, with a lower rate of catheter loss over time (*p* < 0.001, log-rank test). Kaplan–Meier curves are shown in Figure 2.

Table 1 summarizes the laboratory parameters at admission, within 48–72 h of antibiotic administration, and on the last follow-up day (discharge or death) for groups. No meaningful differences were reported between the two groups in C-reactive protein (CRP) and WBC levels on admission or at the last follow-up. However, within 48–72 h of antibiotic administration, Group 1 showed significantly lower CRP levels (*p* = 0.013), whereas a delayed CRP response to antibiotic therapy was noted in Group 2. No patient exhibited fever at 48–72 h after treatment initiation. The frequency of catheter revision (*p* < 0.0001) was lower in Group 1 compared to Group 2.

Overall, the mortality rate was 5.6%. The antibiotic lock protocol achieved a success rate of 88.9%, with clinical resolution observed in 24 of 27 catheter-related bacteremia episodes. The success rates varied by pathogen: 86% (6/7) for GNB infections and 90% (18/20) for GPB infections. Among patients receiving daily antibiotic lock therapy (*n*:7), the success rate was 86%, while those receiving the therapy every three days (*n*:20) had a success rate of 90%.

## 3. Discussion

Long-term intravascular catheters pose a risk of CRBSIs, either at the catheter–tissue contact area or via the luminal access route. The latter typically occurs via contamination of the catheter hub or, less frequently, through contaminated infusates. Causative microorganisms are predominantly derived from the patient’s own skin flora or may be introduced by healthcare personnel due to non-adherence to sterile techniques and inadequate hand hygiene practices [10]. The catheter lumen provides an isolated environment that facilitates bacterial adhesion, proliferation, and biofilm formation, shielded from the host’s immune defenses. The glycocalyx matrix of the bacterial biofilm further enhances microbial survival by providing significant protection against short-term exposure to antimicrobial agents, thereby making the salvage of infected catheters particularly challenging [12].

In this study, CRBSI cases were evaluated over a five-year span at a tertiary care center. The treatment was successfully achieved in approximately 52.1% of CRBSI episodes, with this rate increasing to 88.9% in cases treated with ALT. Several recent studies have demonstrated that the use of ALT alongside appropriate systemic antimicrobial treatment effectively cleared bacteremia and enabled catheter retention in up to 93% of cases [13,14,15]. Rijnders et al. [14] also reported comparable success, showing that the use of antibiotic lock therapy reduced the failure rate in CRBSI treatment from 57% to 33%. Additionally, a study by O’Horo et al. [16] analyzed studies comparing ALT combined with systemic antibiotic therapy versus systemic therapy alone, demonstrating that the combination significantly improved catheter salvage rates (OR 0.2, 95% CI: 0.10–0.39). The median duration of treatment was reported as 14 days. The analysis revealed no significant connection between ALT success and patient sex, age, underlying conditions (diabetes), or catheter type. In the literature, Tejwani et al. [17] reported similar findings regarding the efficacy of ALT, noting that the success rate was independent of epidemiological factors.

In our study, similar to the existing literature, ALT was most applied in hemodialysis patients. Additionally, *Coagulase-negative staphylococci* were identified as the predominant causative agents [2,18]. In our study, the cure rate for CoNS reached up to 92.9% in Group 1, which is consistent with previous reports [13]. Vancomycin was the most frequently used agent for ALT in the treatment of CoNS infections. In the study by Blanco-Di Matteo et al. [19], which evaluated the effectiveness of various ALT agents in hemodialysis patients, Daptomycin locks achieved the highest eradication rates for CoNS from intravascular catheters, while Vancomycin demonstrated a lower success rate of 61.5%. In our cohort, we had one patient who received a Daptomycin lock, and the treatment was successful. Notably, our Vancomycin lock success rate was higher than that reported by Blanco-Di Matteo et al. Differences in patient populations, treatment protocols, antimicrobial concentrations, or follow-up durations may have contributed to the discrepancies observed across studies. Unlike our reactive approach, which focused on treating symptomatic catheter-related infections, Blanco-Di Matteo et al. [20] demonstrated the effectiveness of a pre-emptive strategy based on routine quantitative blood cultures to detect critical catheter colonization and initiate ALT before clinical infection emerged. Their findings suggest that early intervention targeting immature biofilms may improve eradication rates and reduce the progression to bloodstream infection.

GNB were identified in 33.3% of cases. Although all Gram-negative bacteremia episodes in our cohort were classified and treated as primary CRBSI, and episodes with documented alternative infection sources were excluded, distinguishing true primary CRBSI from secondary catheter colonization in a retrospective analysis remains inherently challenging. It is therefore possible that some patients had subclinical or undetected infection foci at presentation. The overall treatment success rate for GNB infections was 40%, and increased markedly to 85.7% in cases where ALT was added to the treatment regimen. In cases of *S. aureus* infection, the overall success rate was 25%, with favorable outcomes observed only in patients who received both intravenous antibiotics and ALT. Two such patients in our cohort had *S. aureus* bacteremia. Neither exhibited tunnel infection, pocket infection, severe sepsis, septic shock, endocarditis, septic thrombophlebitis, osteomyelitis, nor any other form of complicated infection. In both cases, catheter replacement was not feasible. The patients were closely monitored, and ALT was continued as follow-up blood cultures remained negative. The study addressing long-term catheter-related bacteremia due to Gram-negative bacilli reported a 95% cure rate in patients receiving antimicrobial lock therapy [1]. Another study also demonstrated that catheter salvage rates were higher in infections caused by Gram-negative bacilli compared to coagulase-negative staphylococci, with the lowest success rates observed in cases involving *S. aureus* and yeasts. Most recent studies indicate that ALT might be unsuitable for patients diagnosed with *S. aureus* CRBSIs [17,21,22].

Although our study did not identify any cases of resistance associated with ALT, repeated administration of antimicrobial agents within the catheter lumen may pose a theoretical risk of selecting resistant organisms over time. The study by Blanco-Di Matteo et al. [19] also reported an increase in CoNS isolates with higher MIC values among patients with recurrent critical catheter colonization who were treated with ALT, underscoring this potential risk. Antimicrobial resistance remains a growing global health challenge, and even localized interventions like antimicrobial lock therapy should be implemented with careful consideration of long-term ecological impact.

In our cohort, the median time to obtain follow-up blood cultures in the ALT group (Group 1) was 110 h, exceeding the 48–72 h window generally recommended in the literature [11]. By contrast, the non-ALT group (Group 2) adhered to the conventional 48–72 h interval. Specifically, 21 cases in Group 1 and only 4 cases in Group 2 had follow-up cultures drawn after 72 h. This interval is thought to have been extended in Group 1 to more accurately evaluate culture clearance kinetics and the true impact of antibiotic-lock therapy on bacteremia resolution following pathogen identification.

CRP is an acute-phase reactant that increases in response to inflammation. Although specific studies directly examining the relationship between ALT and CRP levels are limited, the existing literature suggests that CRP can serve as a valuable marker for monitoring the response to antibiotic therapy and guiding treatment strategies [23,24]. In our study, lower CRP levels were observed in the IV + ALT group during treatment 48/72 h, which can be interpreted as an indication of a more rapid resolution of the inflammatory response. This may reflect early treatment response; however, this observation should be interpreted cautiously. Further prospective and multicenter studies are warranted to confirm the prognostic significance of CRP trends in patients receiving ALT and to better guide clinical decision-making.

This study has some limitations. First, it was conducted in a single center, which may limit the external validity of the findings. Additionally, the retrospective design and relatively small sample size, particularly the low number of GNB cases, may reduce the statistical power and limit the generalizability of the results to broader populations and diverse clinical settings. Using retrospective medical records as the main data source may have introduced bias or missing information, especially regarding patients’ medical histories, follow-up blood cultures, and recovery progress. Furthermore, there was no standardized protocol for determining the frequency of antimicrobial lock therapy; decisions were made at the treating physician’s discretion based on individual clinical assessment. Lastly, the absence of long-term follow-up data restricted our ability to evaluate the sustained effectiveness and durability of treatment outcomes over time.

## 4. Materials and Methods

### 4.1. Study Design and Setting

The retrospective study was carried out at an 831-bed tertiary care center, functioning as a regional hospital, with a total of 70 intensive care unit (ICU) beds, including 7 pediatric ICU beds, between January 2019 and December 2024. Patients aged ≥18 years diagnosed with CRBSIs who had long-term indwelling catheters used for hemodialysis and/or chemotherapy, and for whom catheter replacement posed significant clinical challenges, were enrolled. Participants were categorized into two groups based on their treatment regimens: those receiving intravenous (IV) antibiotics combined with ALT (Group 1), and those receiving IV antibiotics alone (Group 2). Antibiotic lock solutions were prepared by combining an antibiotic agent, cefazolin, ampicillin, vancomycin, or daptomycin for Gram-positive bacteria (GPB), and gentamicin for Gram-negative bacteria (GNB) using concentrations between 2 and 10 mg/mL in combination with a heparin solution [8,10,11] (Table 3). Five milliliters of ALT solution were instilled into all catheter lumens following each dialysis session and were replaced every 24 or 72 h for a duration of 7–14 days, except in two cases where the therapy was extended to one month. Each patient underwent an assessment by a physician, and the treatment plan was determined by the primary healthcare provider. If surveillance blood cultures detected the same microorganism and catheter removal was documented during treatment due to infection, or if mortality occurred, the treatment was considered unsuccessful. Catheter salvage is defined as the successful preservation of the catheter without recurrence of infection during the treatment period.

### 4.2. Participants

Individuals who (1) were aged 18 and above, (2) were diagnosed with catheter-related bacteremia, (3) did not have exit-site or tunnel infections, (4) were followed up in our clinic, and (5) were treated between January 2019 and December 2024 were considered eligible for inclusion. Cases were ruled out if they (1) were younger than 18 years old, (2) had short-term indwelling catheters, or (3) had an alternative focus of infection. Patient demographics, pathogen distribution, administered antibiotic regimens, duration of treatment, laboratory parameters, microbiological eradication, clinical improvement, and mortality rates were evaluated between the groups. CRBSIs were diagnosed based on the international guidelines for catheter-related infection recommendations [11], including the presence of clinical signs of infection, positive peripheral blood cultures with the same organism isolated from the catheter tip or differential time to positivity (DTP), and the absence of an alternative infection focus.

### 4.3. Data Collection

Demographic details, clinical characteristics, and laboratory findings of the participants were documented using a uniform data collection sheet, utilizing electronic health records. Laboratory values were assessed at the time of admission, within 48/72 h of antibiotic administration, and on the last follow-up day (discharge or death).

Comorbidities, including hypertension (HT), diabetes mellitus (DM), chronic heart disease (CHD), chronic obstructive pulmonary disease (COPD), malignancy, and duration of antibiotic therapy, were recorded. Additionally, routine blood examinations, encompassing complete blood counts, serum biochemical tests, C-reactive protein (CRP) levels, procalcitonin measurements, and culture tests, were recorded. Patients were included more than once if the interval between two CRBSI episodes exceeded three months. The identification of microorganisms was performed using the VITEK 2 automated system (BioMérieux, Marcy- l'Etoile, France).

The study’s primary endpoint was clinical success, defined as the resolution of infection-related signs and symptoms, negative follow-up blood cultures, and catheter retention without recurrence of infection during the treatment period, and patient survival. Secondary endpoints included catheter removal, in-hospital all-cause mortality, and catheter survival time.

### 4.4. Statistical Analysis

Categorical variables were expressed as frequencies and percentages, while continuous variables were summarized as mean ± standard deviation or median with interquartile range (Q1–Q3), as appropriate. The normality of continuous variables was evaluated using the Kolmogorov–Smirnov test. Comparisons between categorical variables were performed using the Chi-square test or Fisher’s exact test, where applicable. For continuous variables, the student’s *t*-test or the Mann–Whitney U test was used based on the distribution of the data. Kaplan–Meier survival analysis was conducted to assess catheter retention over time. Intergroup comparisons were performed using the log-rank test. Statistical analyses were conducted using SPSS software, version 25.0 (IBM Corp., Armonk, NY, USA). A two-sided *p*-value of less than 0.05 was considered statistically significant.

## 5. Conclusions

Antimicrobial lock therapy may be considered a treatment option for managing CRBSIs, particularly in cases where removal of the implantable catheter is not feasible, as it may support catheter preservation. Further studies are needed to optimize lock therapy regimens for different pathogens and patient profiles.

## Figures and Tables

**Table 1:** Patient demographics, clinical features, and laboratory parameters according to treatment groups.

	Group 1 (*n*, %)	Group 2 (*n*, %)	Overall (*n*, %)	*p*
Age (Sd)	66.4 (13.7)	66.2 (17.1)	66.3 (15.4)	0.951
Sex (Female)	8 (29.6)	15 (55.6)	23 (42.6)	0.054
Presence of comorbidity				
Hypertension	16 (59.3)	17 (63)	33 (61.1)	0.78
Diabetes	9 (33.3)	12 (44.4)	21 (38.9)	0.577
CCI median (Q1–3)	6 (5–7)	6 (4–7)	6 (4–7)	0.700
Fever	9 (33.3)	16 (59.3)	25 (46.3)	0.056
Follow up				
Duration of treatment (days), median (Q1–3)	14 (10–14)	14 (12–14)	14 (10–14)	0.599
Catheter days, median (Q1–Q3)	426 (129.5–723)	180 (30–420)	240 (84.5–588)	0.052
Time to follow-up culture (h), median (Q1–Q3)	110 (96–120)	33.5 (0–120)	96 (44.5–120)	<0.0001
Laboratory Parameters				
GPB	20 (74.1)	16 (59.3)	36 (66.7)	0.248
GNB	7 (25.9)	11 (40.7)	18 (33.3)	
CRP (mg/L, normal range: 0–5) On admission	90 (159–56)	122.5 (69.5–158.5)	123 (59.8–173.3)	0.457
CRP (mg/L, normal range: 0–5) (48/72 h)	69.9 (57.4)	119.5 (75.8)	91.6 (69.9)	0.013
CRP (mg/L, normal range: 0–5) (discharge/death)	15 (9–40)	20.5 (11–65)	16 (9–46)	0.291
WBC counts (×10^9^/L, normal range: 4–10) on admission	8509.4 (3247.6)	11,323.3 (4713)	9916.4 (4252.9)	0.014
WBC counts (×10^9^/L, normal range: 4–10) (48/72 h)	7310.7 (3039.9)	9239 (5248.2)	8154.4 (4215.1)	0.117
WBC counts (×10^9^/L, normal range: 4–10) (discharge/death)	6308.1 (3054.8)	7262.7 (2869.2)	6649 (2990.5)	0.328
Outcome				
Positive follow-up culture	3/27 (11.1)	2/10 (20)	5/37 (13.5)	0.482
Catheter removal	3/27 (11.1)	18/21 (85.7)	21/48 (43.8)	<0.0001
In-hospital mortality	0 (0)	3/27 (11.1)	3/54 (5.6)	0.236

CCI: Charlson comorbidity index; GNB: Gram-negative bacteria; GPB: Gram-positive bacteria; WBC: white blood cell; CRP: C-reactive protein. A positive follow-up culture is defined as the detection of microbial growth in blood cultures obtained during treatment, indicating persistent infection.

**Table 2:** Distribution of antibiotics used in catheter-related bloodstream infections.

	Group 1 (*n* = 27, %)	Group 2 (*n* = 27, %)
Ampicillin	3 (11%)	1 (3.7%)
Cephalosporin	5 (18.5%)	7 (25.9%)
Cefazolin	3 (11%)	6 (22.2%)
Ceftriaxone	2 (7.4%)	1 (3.7%)
Carbapenem	4 (14.8%)	6 (22.2%)
Aminoglycoside	1 (3.7%)	4 (14.8%)
Glycopeptide	12 (44.4%)	12 (44.4%)
Vancomycin	11 (41%)	6 (22.2%)
Teicoplanin	1 (3.7%)	6 (22.2%)
Lipopeptide	2 (7.4%)	0 (0%)
Others	0 (0%)	2 (7.4%)

Group 1: ALT + systemic therapy (*n* = 27), Group 2: systemic therapy only (*n* = 27). Ampicillin was preferred for cases with *Enterococcus* spp.; cefazolin was used for *Staphylococcus aureus* and *Coagulase-negative Staphylococci* (CoNS); ceftriaxone was administered in infections caused by *Klebsiella* spp. And *Escherichia coli*. Carbapenems were used in *Enterobacter cloacae*, *Serratia marcescens*, *Klebsiella* spp., and *Pseudomonas aeruginosa* cases. Aminoglycosides were preferred in *Serratia marcescens*, *Escherichia coli*, and *Pseudomonas aeruginosa* infections. Glycopeptides were used for *Staphylococcus aureus* and CoNS, and lipopeptide was used for CoNS cases.

**Table 3:** Antimicrobial lock solution regimens used in the study.

Antimicrobial	Concentration (mg/mL)	Heparin Concentration (UI/mL)
Vancomycin	2.5	2500
Ampicillin	10	3000
Cefazolin	5	2500
Daptomycin	5	2500
Gentamicin	5	2500

## Data Availability

The data that support the findings of this study are available from the corresponding author upon reasonable request.

## References

[1] Funalleras G., Fernández-Hidalgo N., Borrego A., Almirante B., Planes A.M., Rodríguez D., Ruiz I., Pahissa A. (2011). Effectiveness of antibiotic-lock therapy for long-term catheter-related bacteremia due to Gram-negative bacilli: A prospective observational study. Clin. Infect. Dis..

[2] Alfieri A., Di Franco S., Passavanti M.B., Pace M.C., Simeon V., Chiodini P., Leone S., Fiore M. (2025). Antimicrobial Lock Therapy in Clinical Practice: A Scoping Review. Microorganisms.

[3] Mayer J., Greene T., Howell J., Ying J., Rubin M.A., Trick W.E., Samore M.H., CDC Prevention Epicenters Program (2012). Agreement in classifying bloodstream infections among multiple reviewers conducting surveillance. Clin. Infect. Dis..

[4] Alwazzeh M.J., Alnimr A., Al Nassri S.A., Alwarthan S.M., Alhajri M., AlShehail B.M., Almubarak M., Alghamdi N.S., Wali H.A. (2023). Microbiological trends and mortality risk factors of central line-associated bloodstream infections in an academic medical center 2015–2020. Antimicrob. Resist. Infect. Control.

[5] Justo J.A., Bookstaver P.B. (2014). Antibiotic lock therapy: Review of technique and logistical challenges. Infect. Drug Resist..

[6] Raad I.I., Fang X., Keutgen X.M., Jiang Y., Sherertz R., Hachem R. (2008). The role of chelators in preventing biofilm formation and catheter-related bloodstream infections. Curr. Opin. Infect. Dis..

[7] Droste J.C., Jeraj H.A., MacDonald A., Farrington K. (2003). Stability and in vitro efficacy of antibiotic-heparin lock solutions potentially useful for treatment of central venous catheter-related sepsis. J. Antimicrob. Chemother..

[8] del Pozo J.L. (2009). Role of antibiotic lock therapy for the treatment of catheter-related bloodstream infections. Int. J. Artif. Organs.

[9] Korbila I.P., Bliziotis I.A., Lawrence K.R., Falagas M.E. (2007). Antibiotic-lock therapy for long-term catheter-related bacteremia: A review of the current evidence. Expert Rev. Anti Infect. Ther..

[10] O’Grady n.P., Alexander M., Burns L.A., Dellinger E.P., Garland J., Heard S.O., Lipsett P.A., Masur H., Mermel L.A., Pearson M.L. (2011). Guidelines for the prevention of intravascular catheter-related infections. Clin. Infect. Dis..

[11] Mermel L.A., Allon M., Bouza E., Craven D.E., Flynn P., O’Grady N.P., Raad I.I., Rijnders B.J., Sherertz R.J., Warren D.K. (2009). Clinical practice guidelines for the diagnosis and management of intravascular catheter-related infection: 2009 update by the Infectious Diseases Society of America. Clin. Infect. Dis..

[12] Bouhrour n., Nibbering P.H., Bendali F. (2024). Medical Device-Associated Biofilm Infections and Multidrug-Resistant Pathogens. Pathogens.

[13] Fortún J., Grill F., Martín-Dávila P., Blázquez J., Tato M., Sánchez-Corral J., García-San Miguel L., Moreno S. (2006). Treatment of long-term intravascular catheter-related bacteraemia with antibiotic-lock therapy. J. Antimicrob. Chemother..

[14] Rijnders B.J., Van Wijngaerden E., Vandecasteele S.J., Stas M., Peetermans W.E. (2005). Treatment of long-term intravascular catheter-related bacteraemia with antibiotic lock: Randomized, placebo-controlled trial. J. Antimicrob. Chemother..

[15] Viale P., Pagani L., Petrosillo N., Signorini L., Colombini P., Macri G., Cristini F., Gattuso G., Carosi G., Italian Hospital and HIV Infection Group (2003). Antibiotic lock-technique for the treatment of catheter-related bloodstream infections. J. Chemother..

[16] O’Horo J.C., Silva G.L., Safdar N. (2011). Anti-infective locks for treatment of central line-associated bloodstream infection: A systematic review and meta-analysis. Am. J. Nephrol..

[17] Tejwani R., Parry M.F. (2011). Antimicrobial lock therapy as an adjunct to management of catheter-related bacteremia: A community hospital experience. Infect. Dis. Clin. Pract..

[18] Schulin T., Voss A. (2001). Coagulase-negative staphylococci as a cause of infections related to intravascular prosthetic devices: Limitations of present therapy. Clin. Microbiol. Infect..

[19] Blanco-Di Matteo A., Garcia-Fernandez N., Aguinaga Pérez A., Carmona-Torre F., Oteiza A.C., Leiva J., Del Pozo J.L. (2023). In Vivo Effectiveness of Several Antimicrobial Locks to Eradicate Intravascular Catheter Coagulase-Negative Staphylococci Biofilms. Antimicrob. Agents Chemother..

[20] Blanco-Di Matteo A., Garcia-Fernandez N., Aguinaga Pérez A., Carmona-Torre F., Oteiza A.C., Leiva J., Del Pozo J.L. (2022). Pre-Emptive Antimicrobial Locks Decrease Long-Term Catheter-Related Bloodstream Infections in Hemodialysis Patients. Antibiotics.

[21] Maya I.D., Carlton D., Estrada E., Allon M. (2007). Treatment of dialysis catheter-related *Staphylococcus aureus* bacteremia with an antibiotic lock: A quality improvement report. Am. J. Kidney Dis..

[22] Freire M.P., Pierrotti L.C., Zerati A.E., Benites L., da Motta-Leal Filho J.M., Ibrahim K.Y., Araujo P.H., Abdala E. (2018). Role of Lock Therapy for Long-Term Catheter-Related Infections by Multidrug-Resistant Bacteria. Antimicrob. Agents Chemother..

[23] von Dach E., Albrich W.C., Brunel A.S., Prendki V., Cuvelier C., Flury D., Gayet-Ageron A., Huttner B., Kohler P., Lemmenmeier E. (2020). Effect of C-Reactive Protein-Guided Antibiotic Treatment Duration, 7-Day Treatment, or 14-Day Treatment on 30-Day Clinical Failure Rate in Patients with Uncomplicated Gram-Negative Bacteremia: A Randomized Clinical Trial. JAMA.

[24] Dias R.F., de Paula A.C.R.B., Hasparyk U.G., de Oliveira Rabelo Bassalo Coutinho M., Alderete J.R.A., Kanjongo J.C., Silva R.A.M., Guimarães N.S., Simões e Silva A.C., Nobre V. (2023). Use of C-reactive protein to guide the antibiotic therapy in hospitalized patients: A systematic review and meta-analysis. BMC Infect. Dis..

